# Thermometers and Barometers

**Published:** 1871-01

**Authors:** 


### THERMOMETERS AND BAROMETERS.

There ought to be a barometer in every family, and a thermometer in every chamber, that every child may be taught their uses, which, if properly explained and with sufficient clearness, will become a fund of amusement, and interesting and intelligent observation for a life-time.

The barometer measures the heaviness of the atmosphere; the heavier it is, the farther down does it press the mercury; the heavier it is, the fuller the air is of water, and the sooner will it rain. When there is but little water in the air, it is very light; each breath we draw has more oxygen in it, and that makes us lively, and cheerful, and light-hearted, without our knowing the cause. On the other hand, if the air is heavy with water or moisture, each breathful of it has that much less oxygen in it, the blood is that much less purified, and we are lifeless, and restless, and depressed.

It is a very pleasant thing to get up in the morning when we have a pleasant visit or excursion in contemplation, to know that it will not rain that day; and many a time it will save a best dress or best bonnet to be told by the silent friend that it will certainly rain before night. But to be thus informed, the height of the mercury should be marked at night, and there is a convenient arrangement for that purpose; if, on rising in the morning, the mercury has fallen an inch, it will certainly be followed by rain or storm before night; if, on the other hand, it has risen largely, it will clear away in a few hours, although it is raining at the instant.

The uses of the thermometer are to indicate the temperature of our chambers, and to warn us beforehand of the state of the out-door air, that we may adapt our clothing to that day's climate.

The Duke of Wellington's nose was his thermometer, and it was about the longest and biggest we ever saw; he never failed to hoist the window and put his head outside the first thing every morning, "sniff" the air, then give his servant directions what clothing to lay out for him that day. Many a life has been lost by not being dressed warm enough on going out for the day, in consequence of a great change in the weather during the night; for it requires a day or two for the cold "to get into the house." On the other hand, if it suddenly changes to a much greater warmth during the night, thirty or forty degrees warmer, and we go out with the clothing of the previous day, we are oppressed, and change to a lighter dress, and a bad cold is taken. It is never safe to change to a lighter dress, after having dressed in the morning; if we want to "leave off" clothing, let that be at the first dressing, and at no other time. This observance alone will prevent much sickness during a life-time.

In summer time, if we sit in a room of sixty-eight degrees, the system will be certainly and quickly chilled; but in midwinter a room of sixty-eight degrees will feel almost "suffocating," especially if entered soon after a brisk out-door walk. Ordinarily a church feels comfortably warm in winter, if the thermometer stands at sixty-five in the centre of the building, and about five feet from the floor.

